# High efficiency penetration of antibody-immobilized nanoneedle thorough plasma membrane for in situ detection of cytoskeletal proteins in living cells

**DOI:** 10.1186/s12951-016-0226-5

**Published:** 2016-11-03

**Authors:** R. Kawamura, K. Shimizu, Y. Matsumoto, A. Yamagishi, Y. R. Silberberg, M. Iijima, S. Kuroda, K. Fukazawa, K. Ishihara, C. Nakamura

**Affiliations:** 1 Biomedical Research Institute, National Institute of Advanced Industrial Science and Technology, Central5 1-1-1 Higashi, Tsukuba, Ibaraki 305-8565 Japan; 2 Department of Biotechnology and Life Science, Tokyo University of Agriculture and Technology, 2-24-26 Naka-cho, Koganei, Tokyo, 184-8588 Japan; 3 Department of Biomolecular Science and Reaction, The Institute of Scientific and Industrial Research, Osaka University, 8-1 Mihogaoka, Ibaraki, Osaka, 567-0047 Japan; 4 Department of Materials Engineering, School of Engineering, The University of Tokyo, 7-3-1 Hongo, Bunkyo-ku, Tokyo, 113-8654 Japan

**Keywords:** Nanoneedle, Atomic force microscopy, Cytoskeleton, Intermediate filament, Single cell analysis, Mechanobiology

## Abstract

**Background:**

The field of structural dynamics of cytoskeletons in living cells is gathering wide interest, since better understanding of cytoskeleton intracellular organization will provide us with not only insights into basic cell biology but may also enable development of new strategies in regenerative medicine and cancer therapy, fields in which cytoskeleton-dependent dynamics play a pivotal role. The nanoneedle technology is a powerful tool allowing for intracellular investigations, as it can be directly inserted into live cells by penetrating through the plasma membrane causing minimal damage to cells, under the precise manipulation using atomic force microscope. Modifications of the nanoneedles using antibodies have allowed for accurate mechanical detection of various cytoskeletal components, including actin, microtubules and intermediate filaments. However, successful penetration of the nanoneedle through the plasma membrane has been shown to vary greatly between different cell types and conditions. In an effort to overcome this problem and improve the success rate of nanoneedle insertion into the live cells, we have focused here on the fluidity of the membrane lipid bilayer, which may hinder nanoneedle penetration into the cytosolic environment.

**Results:**

We aimed to reduce apparent fluidity of the membrane by either increasing the approach velocity or reducing experimental temperatures. Although changes in approach velocity did not have much effect, lowering the temperature was found to greatly improve the detection of unbinding forces, suggesting that alteration in the plasma membrane fluidity led to increase in nanoneedle penetration.

**Conclusions:**

Operation at a lower temperature of 4 °C greatly improved the success rate of nanoneedle insertion to live cells at an optimized approach velocity, while it did not affect the binding of antibodies immobilized on the nanoneedle to vimentins for mechanical detection. As these experimental parameters can be applied to various cell types, these results may improve the versatility of the nanoneedle technology to other cell lines and platforms.

**Electronic supplementary material:**

The online version of this article (doi:10.1186/s12951-016-0226-5) contains supplementary material, which is available to authorized users.

## Background

**Fig. 1:**
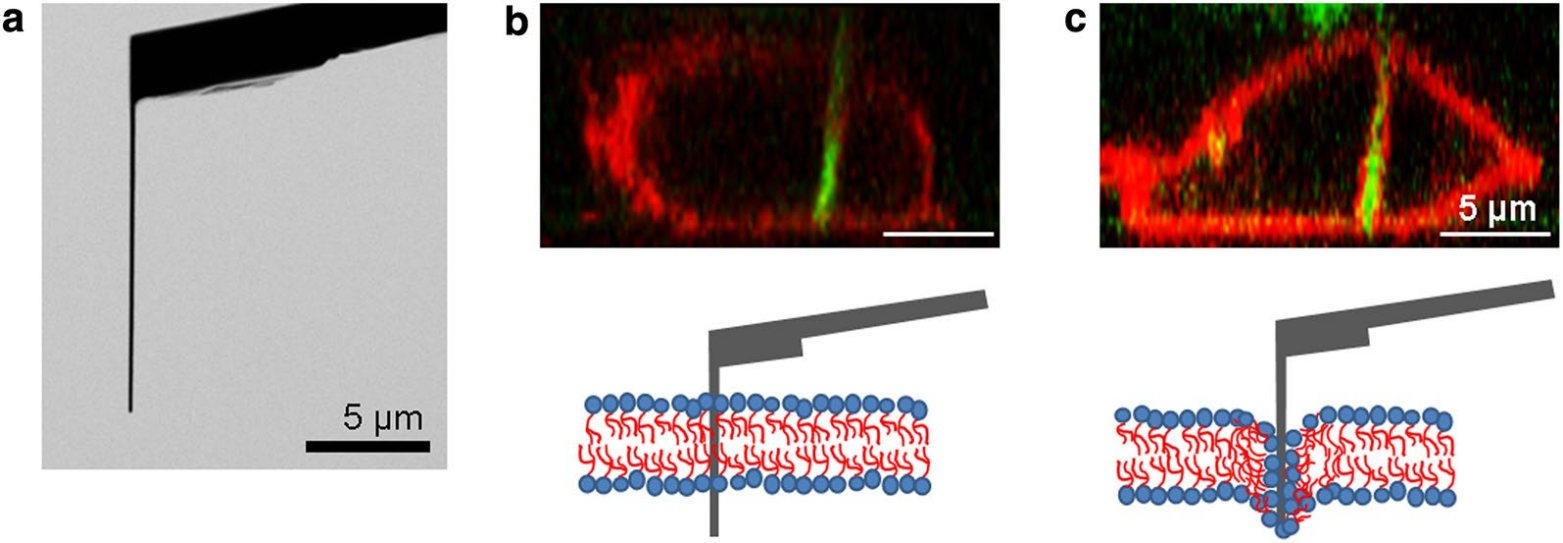
The penetration of nanoneedle through cellular membrane. **a** Scanning ion microscopy (SIM) image of an AFM cantilever type nanoneedle which was obtained immediately after the fabrication by FIB. The nanoneedle was approached to adhering cell to penetrate plasma membrane. **b** Side views reconstructed from confocal fluorescence images of CLSM are showing the successful penetration. **c** Side views reconstructed from confocal fluorescence images of CLSM are showing the failed case

It is well known that the dynamic structure and cell mechanics are realized by the cooperative assembly/disassembly of cytoskeletal elements, which consist of actin filaments, microtubules, intermediate filaments and their related proteins [1, 2]. Dynamic structure alterations have been found to play pivotal roles in various biological phenomena, including developmental process and cancer metastasis [3, 4]. In particular, the role of the over 50 proteins constituting intermediate filaments have remained vastly unknown, while reported to be linked to cancer cell migration in recent studies [5–9]. We have been developing a method for intracellular diagnosis of target cells by inserting a monolithic nanoneedle under the control of an atomic force microscopy (AFM) system [10–13]. This nanoneedle technique allows mechanical detection of target molecules using force spectroscopy, a unique approach for detecting intracellular molecules in real-time [13–15]. An ultra-thin rod-shape with high-aspect ratio (Fig. 1a) allows for efficient insertion through the plasma membrane and into the cytosol of various cell types with minimal cell damage, while modification to the nanoneedle with antibodies allows for specific binding of the nanoneedle to intracellular cytoskeletal protein targets that can be quantified during needle evacuation from the cell. In a recent research, fabrication of nanoneedles in arrayed form was realized and the application to thousands of cells simultaneously was successfully demonstrated [16]. Therefore, development of nanoneedle-based techniques can lead to establishing of new technologies for mechanical analysis of the dynamic structure of cytoskeletal proteins in vivo and identification of the relevant proteins, in addition for its applications in cell sorting by the direct detection of the cytoskeleton. However, successful insertion of nanoneedles through the plasma membrane and into the cytosol remains challenging, and is greatly dependent on cell type, as well as other factors. Hence, optimizing insertion rates will significantly improve the future applicability of this technology [12, 17].

One of the major reasons for nanoneedle insertion failure can be attributed to the soft and fluidic nature of cellular membranes, which lead to their flexible deformation and an unsuccessful penetration [10, 12]. As membrane fluidity is determined also by the dynamics of lipid molecules that constitute the membrane, and these are affected by temperature, we set here to investigate the effect of temperature, as well as approach velocity, on the success rate of nanoneedle penetration.

In this paper, we test a possibility to improve nanoneedle insertion efficiency by verifying the effect of nanoneedle approaching velocity and operating temperature, focusing on membrane fluidity. With the optimization of these parameters to a variety of cell types, applications of the nanoneedle technology can be expanded to any kind of intracellular investigations in biological and biomedical research.

## Results and discussion

### Evaluation of nanoneedle penetration into live cells

Insertion of an AFM cantilever-type nanoneedle into the cytosol of live cells has been originally observed during AFM force spectroscopy measurements, evident from a sharp force relaxation peak in the force-distance curve during cell indentation. Successful insertion of nanoneedles into cells has been further confirmed in other studies investigating molecular functions in the cytosol with the use of FRET or molecular beacon [18–20]. Nanoneedle insertion through the plasma membrane has also been directly visualized with use of confocal laser scanning microscope (CLSM) (Fig. 1b) [10]. In some cases, such as in some cell lines and under certain growth conditions, the higher deformability of the plasma membrane prevents a successful penetration of the nanoneedle into the cell. In these cases, the membrane can be observed surrounding the nanoneedle periphery as it indents into the cell, indicating the lack of actual penetration through the membrane (Fig. 1c). However, that is not always clearly visible. A better way to ascertain successful insertion of the nanoneedle through the plasma membrane is to modify the nanoneedle with antibodies specific for intracellular cytoskeletal proteins and observe the force-distance curves, looking out for significant unbinding events during nanoneedle retraction [13]. Here, this force-detection method was employed for direct and prompt evaluation of penetration events, extending the target protein to vimentin, rather than those previously reported such as actin, microtubule and nestin [13–15].

### Effect of approaching velocity on nanoneedle penetration

**Fig. 2:**
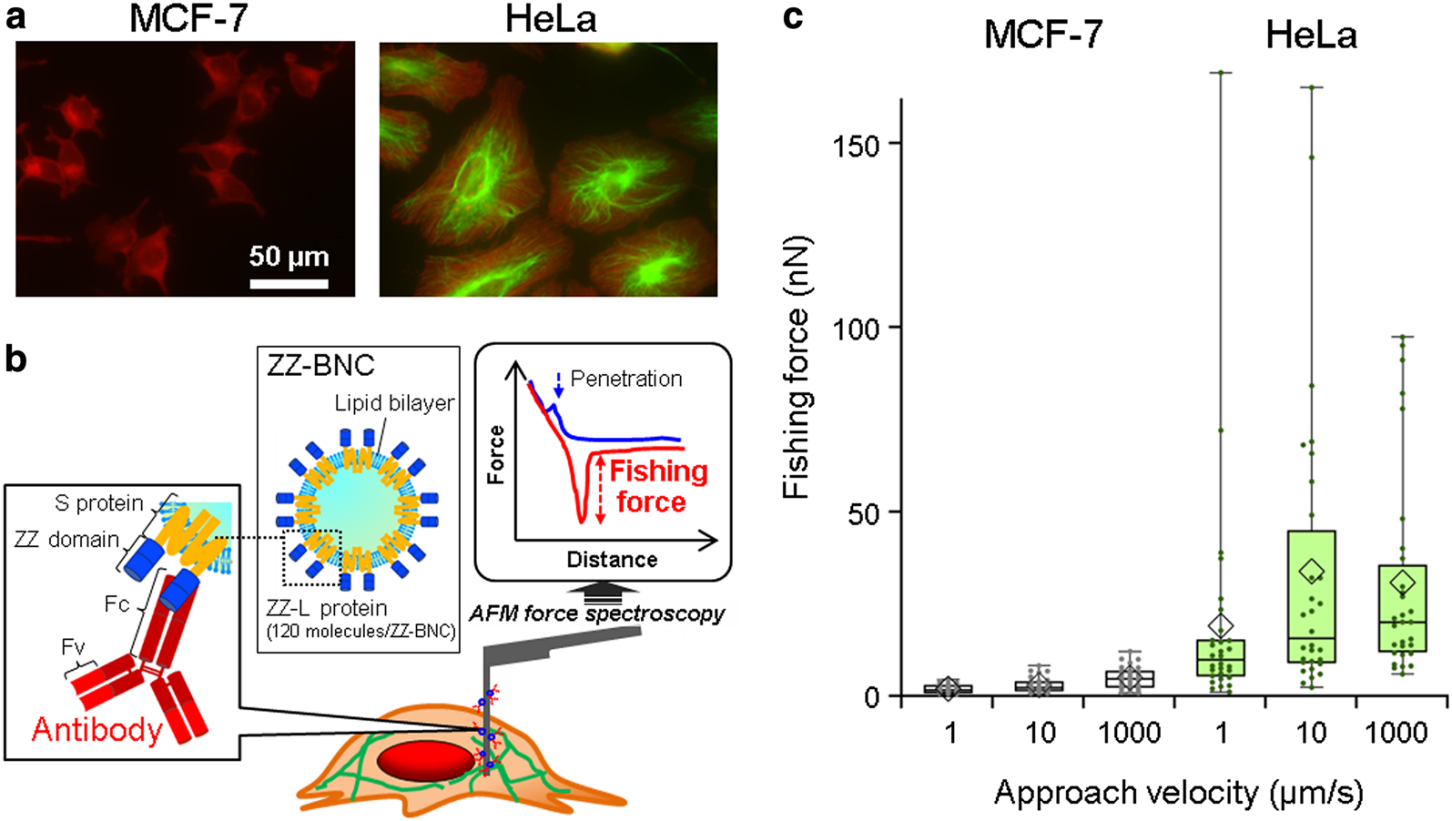
The effect of nanoneedle approach velocity on penetration through the plasma membrane. **a** Fluorescent microscopy of MCF-7 and HeLa cells. For both cell types, actin and vimentin were stained with rhodamine phalloidin and anti-vimentin antibody visualized with secondary anti-mouse IgG labeled with Alexa 488, respectively. **b** Set up of a nanoneedle modified with anti-vimentin antibodies via ZZ-BNC, and the detection of fishing force. **c** Fishing forces detected for MCF-7 and HeLa cells as vimentin-negative and -positive control, respectively. The *horizontal line* within the *box* indicates the median, *boundaries of the box* indicate the 25th- and 75th-percentile, and the *whiskers* indicate the highest and lowest values of the results. Approach velocity was at 1, 10, or 1000 µm/s, while retracting velocity fixed at 10 µm/s. This measurement was performed at room temperature (25 °C). Plots by *small dot* and *diamond-shaped symbol* represent the fishing force and the average fishing force, respectively

Vimentin-detection experiments using anti-vimentin-antibody-modified nanoneedles, unbinding forces (‘fishing forces’) measured for the vimentin-positive HeLa cells were significantly higher than those measured for the vimentin-lacking MCF-7 cells (Fig. 2a). Low fishing forces, such as these detected for MCF-7 cells, are common and are due to non-specific interactions between the nanoneedle and various intracellular components, including the plasma membrane, both in cases of successful and unsuccessful penetration. However, only when the nanoneedle is successfully inserted deep into the cytosol, and only in the presence of the target cytoskeletal protein (vimentin in this case), large fishing forces are detected, indicating true unbinding events between the antibodies on the nanoneedle and the target cytoskeletal proteins (Fig. 2b). Detections of large fishing force for various cytoskeletal proteins including vimentin are proof that antibody-modified nanoneedles penetrate through the cellular membrane and access the cytoskeleton [13–15].

According to our previous reports, the optimal velocity for successful insertion was found to be in the range of 3–10 µm/s, following tests with velocities of up to 1000 µm/s. Considering the fluidity of lipid molecules, which have diffusion coefficient in a range of 0.1–1 µm^2^/s [21], a fast approach velocity seems to cause decrease of apparent membrane fluidity and increases the chance of successful insertion. In this study, we tested approach velocities of up to 1000 µm/s (Fig. 2c), which was the highest velocity possible in our setup. The results show that the highest approach velocity (1000 µm/s) did not show significant increase in fishing force detection of vimentin for the vimentin-positive HeLa cells, compared to that of the normal velocity of 10 µm/s (Fig. 2c; Table 1). The top quartile and the median values in the fishing forces detected for the lowest velocity (1 µm/s), though, was less than that observed in the higher velocity conditions (Fig. 2c, HeLa). Since the lipid membrane may behave in a more fluidic manner at lower approach velocities, it can be speculated that the lipid membrane is less likely to be disrupted in slower approach velocities and will cover the nanoneedles surface, preventing interactions between the nanoneedle-immobilized antibodies and the intracellular vimentin. These tendencies are consistent with our interpretation in which the fast motion of the needle can enhance penetration rate through the soft membrane. The large standard deviation in the fishing forces for vimentin-positive HeLa cell (Table 1) was caused by the small fishing forces detected in the 30 times trials. As vimentins distribute heterogeneously in the cytosol (Fig. 2a), very weak fishing forces will be detected at vimentin-poor sites, while large fishing forces are measured due to multiple specific binding events in vimentin-rich sites of the cytosol. In summary, from the overall results, we concluded best insertion conditions to occur at a velocity of 10 µm/s due to the top quartile in fishing force (Fig. 2c). Since the lipid membrane is ruptured by the nanoneedle on the deflective AFM cantilever that has a spring constant of 0.1–0.4 N/m, the rate condition required for penetration is more properly described by a force-loading rate of 1–4 µN/s.

**Table 1:** Average of the fishing forces detected in MCF-7 and HeLa cells at various approach velocities: analysis on the data set of Fig. 2c

	MCF-7			HeLa		
Approach velocity (µm/s)	1	10	1000	1	10	1000
Fishing force (nN)						
AV	1.8	2.9	4.8	18.9	33.7	30.7
SD	1.2	2.0	2.9	31.8	40.2	28.3
*n*	30	30	30	30	30	30

### Effect of temperature on nanoneedle penetration

**Fig. 3:**
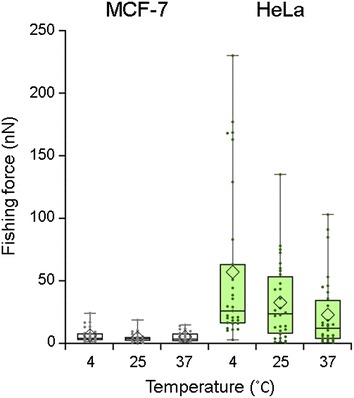
The effect of temperature on the fishing force detection. Fishing forces detected for MCF-7 and HeLa cells at different temperatures with use of anti-vimentin antibody immobilized nanoneedles. The *horizontal line* within the *box* indicates the median, *boundaries of the box* indicate the 25th- and 75th-percentile, and the *whiskers* indicate the highest and lowest values of the results. Velocity of needle approach and retraction was fixed at 10 µm/s. Plots by *small dot* and *diamond-shaped symbol* represent the fishing force and the average fishing force, respectively

Lipids have transition temperature (*T*_c_) at which the fluidity and elasticity drastically changes. As diffusion coefficient of phosphatidylcholine, one of the major components of the cellular membrane, was reported to decrease below 20 °C [21], we expected our nanoneedles to penetrate more efficiently at lower temperatures. Lower temperature conditions of 4 °C were prepared by immersing donut-shaped ice blocks of frozen medium in the dish during force measurements within 15 min (Additional file 1: Figure S1), while higher temperature of 37 °C was maintained by a dish-heating device. Results are shown in Fig. 3 and Table 2. The average fishing force of the vimentin-positive HeLa cells increased more than twice with decrease in temperature from 37 to 4 °C, and the distribution of results became wider at lower temperatures as well; structure and amount of the vimentin were found to be unaffected by the low temperature treatment from immunostaining and western blotting analysis (Additional file 1: Figures S2, S3). Meanwhile, the fishing forces for the vimentin-negative MFC-7 cells remained almost constant throughout the temperature variation. Signal to noise ratio (S/N), which in our case defined as the ratio of fishing force detected for HeLa divided to that detected for MCF-7 cells, was clearly improved by decreasing the temperature to 4 °C, as shown in Table 3. Thus, the low temperature of 4 °C was shown to enhance the detection of higher fishing forces. With decrease in the temperature from 37 to 4 °C, the highest measured fishing forces for HeLa cells increased by a factor of more than 2, suggesting increase in binding events between antibodies and vimentin proteins. This is perhaps due to a decrease in thermal fluctuations of vimentin at lower temperatures, and also due to decrease in contamination of the nanoneedles with amphiphilic lipid molecules.

**Table 2:** Average of the fishing forces detected in MCF-7 and HeLa cells at various temperature: analysis on the data set of Fig. 3

	MCF-7			HeLa		
Temperature (°C)	4	25	37	4	25	37
Fishing force (nN)						
AV	6.3	4.4	5.2	56.9	32.5	22.8
SD	5.4	3.5	4.0	63.8	31.8	28.2
*n*	30	30	30	30	30	30

**Table 3:** The ratio of successful fishing force detection in HeLa cells at low temperature: analysis on the data set of Fig. 3

	4 °C	37 °C
Detection of fishing forces over threshold* (*n* out of 30 trials)	14	9
Detection of fishing force over threshold* (%)	46.7	30.0
Average of fishing force over threshold (nN) ± SD	100.7 ± 69.1	58.8 ± 26.8
S/N**	9.0	4.4

* Threshold equals to (average fishing force of MCF-7 cells as negative control at each temperature) + 4 × (standard deviation)

** Ratio of (average fishing force of HeLa)/(average fishing force of MCF-7)

**Fig. 4:**
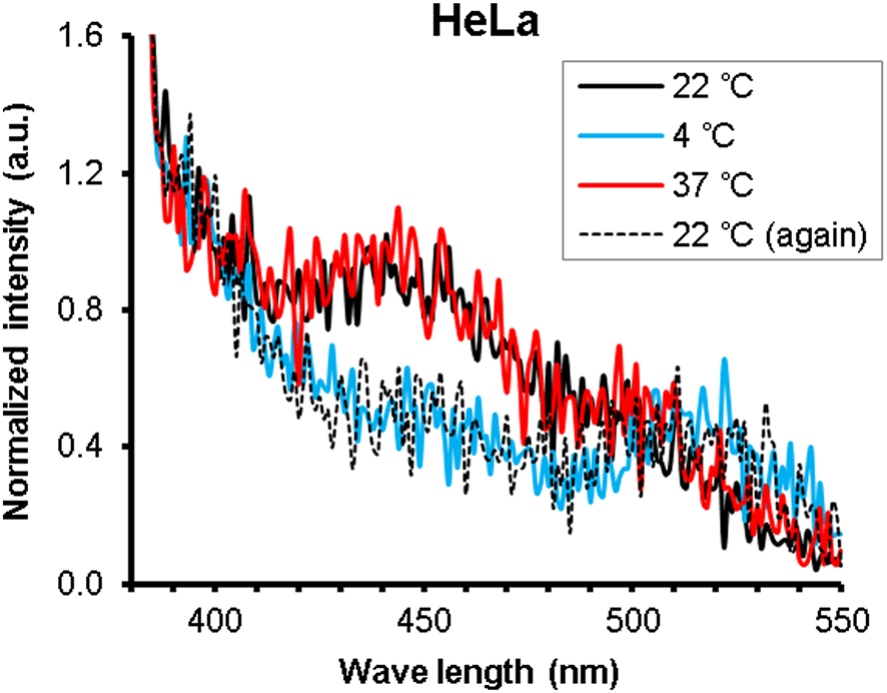
The fluorescence spectra of pyrene. Temperature was varied in the order of 22, 4, 37, 22 °C after treating the cells with pyrene and fluorescence spectra of them were measured. When excimer of pyrene is formed in fluidic membrane, emission peak at 450 nm appears. As the peak at 450 nm disappeared at final 22 °C, local lipid domain seemed to form and allowed to become less fluidic at higher temperature than initial state

To check the change in lipid membrane fluidity, we further analyzed excimer formation of pyrene with interaction to the cellular membrane. As a result, we could see a decrease in the fluorescence intensity at 450 nm for measurements at 4 °C, suggesting that the membrane is less fluidic at the lower temperature (Fig. 4). According to reports on *T*_c_ of the lipid membrane with use of fluorescent probes such as 1,6-diphenyl-1,3,5-hexatriene (DPH) or pyrene, the membrane of HeLa cells have *T*_c_ of ca. 16 °C [22, 23]. This supports the previous reasoning of lowering membrane fluidity at lower temperature.

Stiffness of the plasma membrane was estimated from force-indentation curves during nanoneedle insertion into the cells (Additional file 1: Figure S4). Young’s modulus was calculated by fitting with the Hertz model for initial deformation, to evaluate stiffness of the plasma membrane. Significant differences were not observed in cell stiffness due to temperature changes (Additional file 1: Figure S4) since fluidity of lipid bilayer may not have large contribution to cell stiffness. However, the average cell stiffness under 4 °C condition was higher than that under 37 °C. This tendency is consistent with the decrease in the fluidity (Fig. 4).

From the results for HeLa cells it was suggested that membrane fluidity under low temperature condition would contribute to deeper insertion of nanoneedle into cell. Further validation with other combinations of target intracellular molecules and cell types will be performed in the near future, in order to investigate whether insertion failure is perhaps due to fluidity variations between different cell types. ZZ-BNC, the anchoring agent for the antibody seems to be rigid enough to keep the spherical structure during the temperature change between 5 and 37 °C, since it consists of 80 % protein, 10 % sugar chain and 10 % lipid that derived from endoplasmic reticulum of yeast [24].

In summary, it is reasonable to conclude that lowering membrane fluidity at lower temperatures enhances penetration efficiency, although other possibilities should be considered. In an attempt to consider other possible mechanisms for enhancing fishing force detection, we followed with examining the effect of temperature on vimentin-antibody unbinding forces.

### Lack of temperature effect on antibody-antigen unbinding forces

**Fig. 5:**
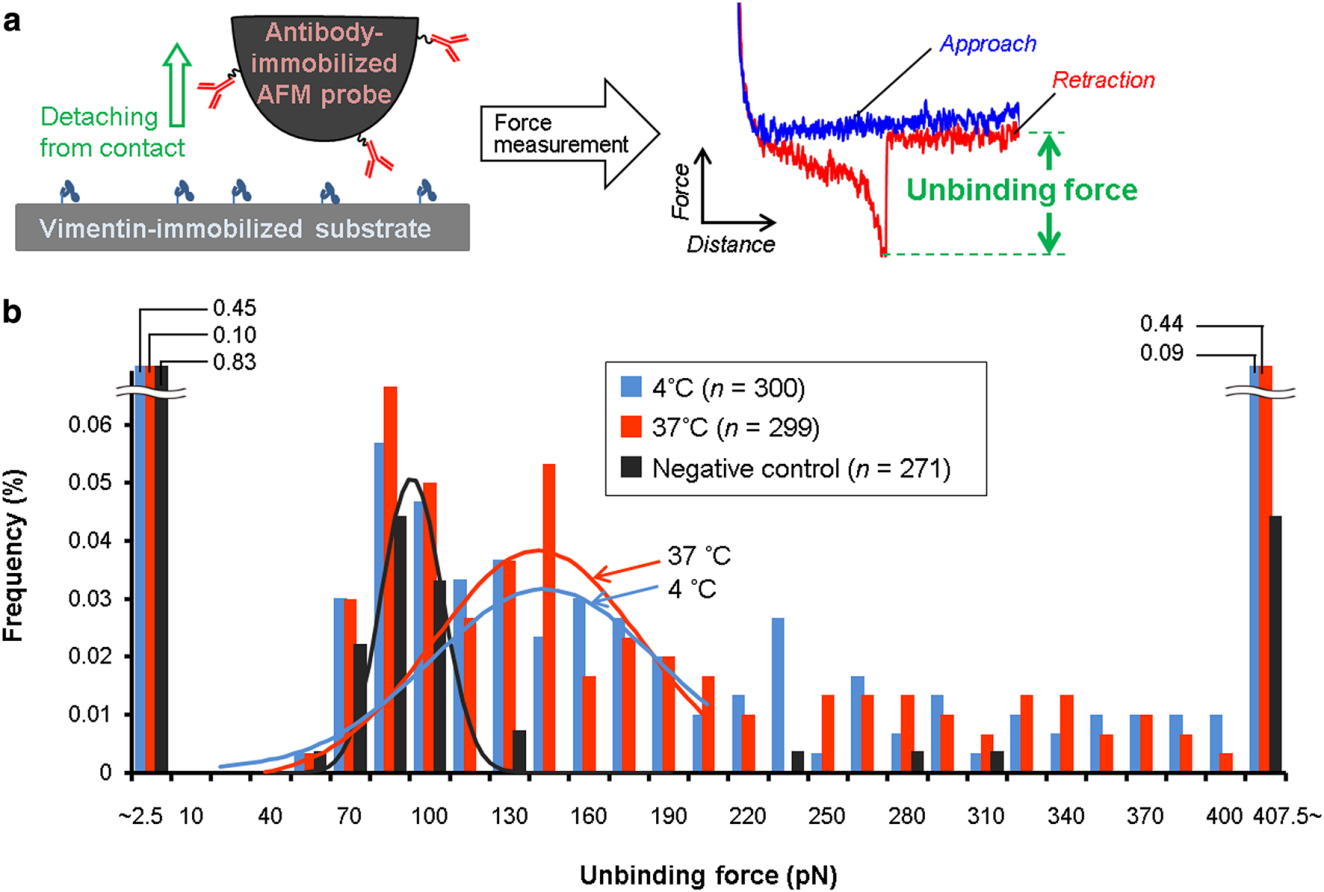
The effect of temperature on antibody-antigen unbinding force. **a** Unbinding forces of antibody-antigen interactions were measured in vitro using anti-vimentin antibody-immobilized AFM probe and vimentin-immobilized substrate. The maximum peak force detected on force curve during cantilever retraction was defined as ‘unbinding force’. **b** Measured unbinding forces were plotted as a histogram for the cases of 4 and 37 °C, and for negative control (no vimentin modification on the substrate). Fitting curves for the cases at 4 and 37 °C on vimentin-coated surface were obtained assuming multiple Gaussian distribution after first peak at around 80 pN, although single peak was indicated in data of 4 and 37 °C omitting the peak of nonspecific interaction at 80 pN and not clear peaks of multiple unbinding. Fitting curve for negative control data was obtained by assuming single Gaussian distribution

In order to verify whether or not temperature has an effect on the unbinding forces between vimentin and anti-vimentin antibodies, unbinding measurements were conducted between standard pyramidal AFM cantilever to which anti-vimentin antibodies were covalently bound, and glass substrate to which vimentin was covalently immobilized on. ‘Unbinding force’ was defined as the peak force detected immediately before going back to the baseline (Fig. 5a). Following contact of the cantilever with the glass surface, unbinding forces were measured at 4 and 37 °C, and with a negative control (no vimentin-bound surface, room temperature) (Fig. 5b, *n* was approximately 300 for each case). For both 4 and 37 °C, the measured mean unbinding force stood on 140 pN, as can be seen form the histograms (Fig. 5b), while it was 80 pN for the non-specific interactions (negative control lacking vimentin on the substrate). Positive skewness of the distribution can be seen around the peak of 140 pN at 4 and 37 °C, while the negative control did not show this skewness. This could be caused by multimodal distributions due to multiple bindings of antibodies and vimentins. Curve fitting assuming multimodal Gaussian distribution derived from multiple bindings did not give a clear peak, although more measurements may lead to identification of secondary and following peaks [25]. The outlying distribution of the forces less than 2.5 nN is probably due to non-specific interactions between the substrate and the tip on which the antibody is hindered by chance due to the flexibility of the linking polymer, while those over 407.5 nN are artefacts that seem to be caused by non-specific interaction with dust impurities. Thus, it was verified that temperature did not have an effect on the strength of interactions between the vimentin and anti-vimentin antibodies. This result supports the interpretation that the lower temperature leads to an increase in fishing forces due to the decrease in membrane fluidity, as suggested above.

## Conclusions

In this study we aimed to optimize the conditions required for the successful insertion of nanoneedles into live cells, for the purposes of intracellular protein detection, drug/protein delivery, or intracellular manipulation. Both approach velocity and temperature influenced to nanoneedle penetration, especially lowering the temperature was found to greatly improve the detection of unbinding forces, suggesting that alteration in the plasma membrane fluidity led to increase in nanoneedle penetration. As phase transition caused by lowering temperature is general effect for cell membrane, this approach may lead to enhanced versatility in the use of nanoneedles, allowing application of this technology various cell types.

## Methods

### MCF-7 and HeLa cells culture

Human breast cancer cells (MCF-7) and cervical cancer cells (HeLa) were maintained in Dulbecco’s modified Eagle’s medium (DMEM; D5546, Sigma-Aldrich) supplemented with 10 % fetal bovine serum (FBS; Life technologies), 2 mM GlutaMAX (35050-061, Life technologies), gentamycin–amphotericin B (10 and 0.25 µg/ml, GA; R-015-10, Life technologies). The cells were subcultured at 80–90 % confluency every 3 days by following procedures. They were treated with 0.25 % trypsin, 0.01 % EDTA for 3 min at 37 °C and diluted with the medium containing FBS. After centrifuging at 170×*g* for 5 min at room temperature, the cells were seeded into cell culture flasks (353108, Becton–Dickinson) or dishes (93040, TPP).

### Force analysis of cell fishing and adhesion with AFM

Nanoneedles were fabricated from pyramidal silicon AFM cantilevers (ATEC-Cont, Nanosensors) and etched to a cylindrical shape of 200 nm in diameter and 10–15 µm in length, using a focused ion beam (SMI500, Hitachi High-Tech Science). Spring constants (*k* = 0.1–0.4 N/m) were determined using the thermal fluctuation method prior to each experiment [26]. The silicon surface was cleaned with oxygen plasma in a plasma asher (200 W, 5 min; JPA300, J-science) and treated with 1 % HF for 1 min. After repeating the plasma (10 min) and 1 % HF treatment once again, the nanoneedle was modified by physical adsorption of 50 µg/ml of ZZ-BNC at room temperature for 1 h; ZZ-BNC is bio-nano capsule based anchor to which Fc domain of antibodies can bind [27–29]. Anti-vimentin antibody (V6630, Sigma-Aldrich) was bound to the ZZ-domain of the ZZ-BNC by incubating with 475 µg/ml antibody in PBS at room temperature for 1 h. The antibody-immobilized nanoneedle was rinsed in prior to the use in cell fishing experiments. Force measurements were carried out using a Nanowizard II BioAFM (JPK Instruments AG) with CellHesion® unit that allows long traveling distance over 100 µm. For the cellular membrane penetration test, nanoneedles were inserted to cells at approach velocity of 1–1000 µm/s with a set point of 40 nN, left to dwell within the cells for 2 s, and then evacuated at 10 µm/s. 10 different sites on each cells were targeted for insertion and 3 cells were tested for each cell type. The nanoneedles used for more than 5 cells were washed with 0.05 % Tween 20 in PBS before the use for next cells. Temperature control of the sample was done by dish heater to manage sample dish temperature at 37 °C and by putting donut-shaped ice block of medium in the dish during a measurement at around 4 °C. All of the measurements at low temperature were finished within 15 min.

For unbinding force measurement of vimentin-antibody interaction in vitro, an MPC polymer was used for surface modification of nanoneedles to allow stable linking by covalent bonds [14, 15]. AFM cantilevers (ATEC-Cont) were cleaned with plasma-asher for 5 min at 200 W and rinsed with ultrapure water and ethanol once for each. After cleaning with plasma asher once again, AFM cantilevers were dipped in a solution of 0.05 wt% MPC polymer in dry ethanol containing 1-butanol (95:5) for 10 min at room temperature, followed by baking at 50 °C for 15 min [14]. Following rinsing with ethanol 3 times and with PBS once, needles were then soaked in 475 µg/ml anti-vimentin antibody solution for 1 h and rinsed with PBS 3-times. The remaining active ester of MPC polymer was killed by treating with 10 mM ethanol amine for 30 min at room temperature and rinsed with PBS twice. Vimentin (His-tagged human, SRP5150, Sigma-Aldrich) immobilization to glass surface was done in the same way as the AFM cantilever using MPC polymer but with vimentin solution of 50 µg/ml. Force measurement by AFM was performed with approach/retraction velocity at 1 µm/s in a force mapping mode in which 10 × 30 times measurement was made within area of 10 µm square on the substrate.

### Imaging of the cells and the needles by microscopy

Fabricated nanoneedles were cleaned with plasma-asher for 5 min at 200 W and rinsed with ultrapure water and ethanol once for each. After clean with plasma asher once again, the nanoneedle was incubated with MPTMS and the exposed thiol group was further modified with Alexa Fluor 488 maleimide (A1025, Life technologies). For fluorescent visualization, cells were transformed to express Keima-Red protein by transfection of pPM-mKeima-Red. Fluorescence image of cells were obtained with use of confocal laser scanning microscopy system (CLSM; FV-300/IX71, Olympus).

### Fluorescence spectroscopy of cells treated with pyrene with temperature variation

Cells were seeded on to collagen coated glass bottom 6-well plate at density of 7 × 10^5^ per well on the day before measurement. After rinsing with PBS, cells were incubated with 1 ml of 15 µM pyrene in a perfusion buffer A (100 mM NaCl, 5 mM KCl, 2 mM MgCl_2_, 2 mM CaCl_2_, 5 mM d-glucose, 50 mM mannitol, 5 mM HEPES/Tris, pH 7.4) supplemented with 0.08 % Pluronic F-127 for 20 min at 25 °C in the dark. After rinsing with the perfusion buffer A twice, the cells were recovered by incubating with 2 ml of Opti-MEM (31985-062, Life technologies) containing 10 mM HEPES (pH 7.4) and EDTA for 30 min. Fluorescence spectroscopy was performed with excitation light wave length of 360 nm for the emission light wave length range of 380–550 nm at each temperature. Each well was measured three times and the obtained data was averaged. The decreasing of medium temperature down to ca. 4 °C was done by exchanging the medium with cold one and waiting for 5 min after adding frozen medium as described in Additional file 1: Figure S1.

## Additional file

**Additional file 1: Figure S1.** Preparation of donut-shaped ice-block of culture medium. **Figure S2.** Validation of vimentin structures after low temperature treatment. **Figure S3.** Validation of vimentin amount after low temperature treatment. **Figure S4.** Stiffness of cells measured during nanoneedle insertion at different temperatures.
