# Supplementary material for: High efficiency penetration of antibody-immobilized nanoneedle thorough plasma membrane for in situ detection of cytoskeletal proteins in living cells
Source: J Nanobiotechnology. 2016 Nov 3;14:74. doi: 10.1186/s12951-016-0226-5 (PMC5094046; doi:10.1186/s12951-016-0226-5)
Supplement: Supplementary file 1 — Additional file 1: Figure S1. Preparation of donut-shaped ice-block of culture medium. Figure S2. Validation of vimentin structures after low temperature treatment. Figure S3. Validation of vimentin amount after low temperature treatment. Figure S4. Stiffness of cells measured during nanoneedle insertion at different temperatures. [file 12951_2016_226_MOESM1_ESM.pdf]

Additional file:

High efficiency penetration of antibody-immobilized nanoneedle thorough plasma membrane for in situ detection of cytoskeletal proteins in living cells

Ryuzo Kawamura[1], Keita Shimizu[2], Yuta Matsumoto[2], Ayana Yamagishi[1], Yaron R. Silberberg[1], Masumi Iijima[3], Shun'ichi Kuroda[3], Kyoko Fukazawa[4], Kazuhiko Ishihara[4] and Chikashi Nakamura[1,2*]

[1] Biomedical Research Institute, National Institute of Advanced Industrial Science and Technology (AIST), Central4 1-1-1 Higashi, Tsukuba, Ibaraki 305-8562, Japan
[2] Department of Biotechnology and Life Science, Tokyo University of Agriculture and Technology, 2-24-26 Naka-cho, Koganei, Tokyo 184-8588, Japan
[3] The Institute of Scientific and Industrial Research, Osaka University, 8-1 Mihogaoka, Ibaraki, Osaka 567-0047, Japan.
[4] Department of Materials Engineering, School of Engineering, The University of Tokyo, 7-3-1 Hongo, Bunkyo-ku, Tokyo 113-8654, Japan
* Corresponding Author: chikashi-nakamura@aist.go.jp

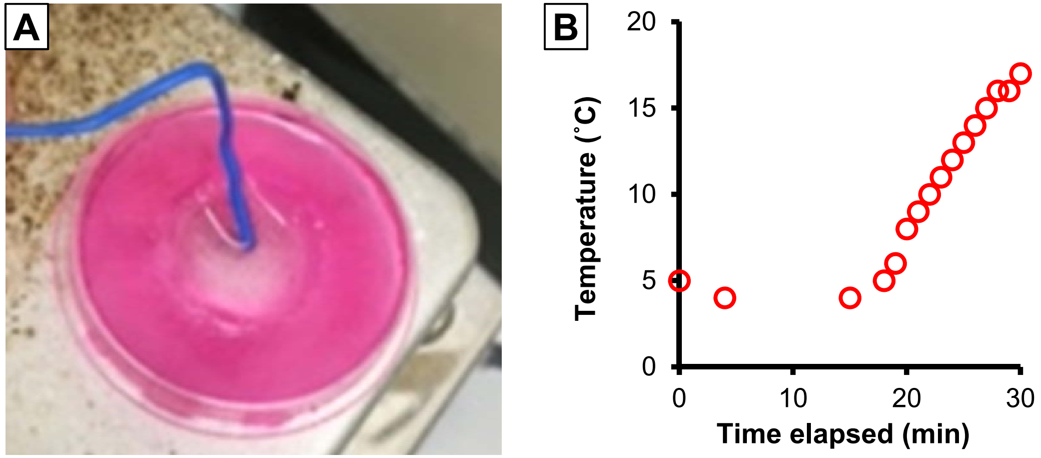

Figure S1. Preparation of donut-shaped ice-block of culture medium. (A) Donut shaped ice block of the culture medium was prepared by freezing in a 35 mm-dish with a molding spacer at the center to have a space for AFM measurements. (B) Until this ice block get completely melt, the temperature of the liquid was kept below 5˚C.

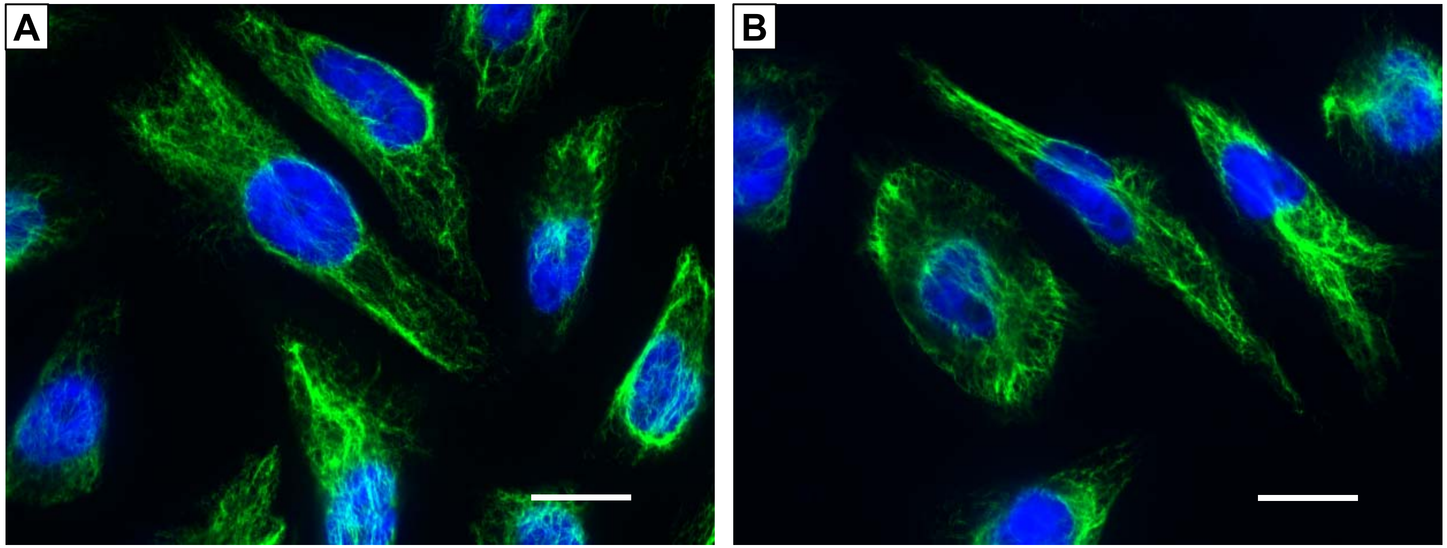

Figure S2. Validation of vimentin structures following low temperature treatment. Vimentin filaments and nuclei of HeLa cells were stained with Alexa 488 (green) and DAPI (blue), respectively. (A) Cells were incubated at less than 5˚C for 15min by adding ice-block of the medium to the dish or (B) kept at 37˚C as negative control. There was no significant change in the pattern nor the amount of vimentin by low temperature treatment. Scale bars are 20 μm.

Methods of cell staining: HeLa cells cultured on a glass bottom dish were rinsed with PBS and fixed with 4% paraformaldehyde for 15 min. After rinsing with PBS 3 times, cells were treated with 0.2% Triton X-100/PBS for 2 min and rinsed with PBS twice. Non-specific interactions were suppressed by blocking with 0.4% bovine serum albumin (BSA)/PBS for 15 min; hereafter rinse was done by 0.4% BSA/PBS. Vimentin was first modified with anti-vimentin antibody (1:1000-dilution, mouse monoclonal antibody, Sigma Aldrich) for 1 h, and labelled with secondary antibody (1:1000-dilution, goat anti-mouse IgG conjugated with Alexa Fluor 488, Invitrogen) after rinsing 3 times. Nuclei were stained using 1 g/ml DAPI (Dojindo) for 30 min followed by rinsing. Cells were observed under confocal laser scanning microscope. The staining process was performed at room temperature. The obtained images were merged using ImageJ software.

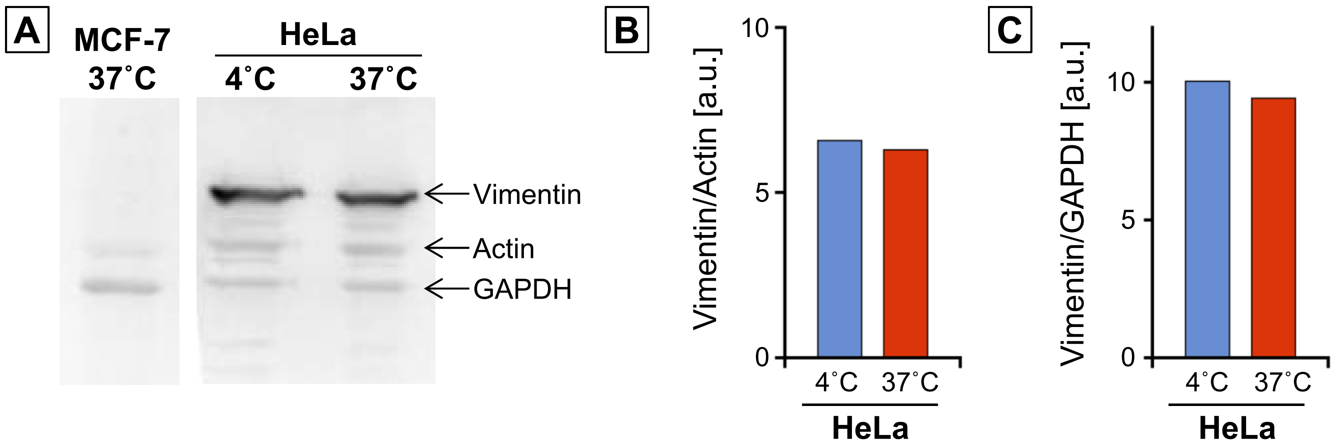

Figure S3. Validation of vimentin amount after low temperature treatment. (A) Proteins expressed in the cells were detected by western blotting. (B) Amount of vimentin was evaluated by densitogram of the western blot and normalized by actin or (C) by GAPDH. No significant difference in vimentin expression was observed following low-temperature treatment.

Methods of western blotting: Cells with or without low temperature treatment (4˚C, for 15 min) were harvested by centrifuge at 70×g for 3 min after PBS rinse and lysed with RIPA buffer supplemented with protease inhibitor. 10 µg of the extracted protein was applied to each lane of 5-20% gradient polyacrylamide gel and separated by SDS-PAGE; protein concentration was measured by using BCA protein assay kit (Pierce). The separated proteins were blotted to PVDF membrane at 30 V for 3 h according to standard semidry method. The membrane was washed with TBS containing 0.1% Tween 20 (TBS-T) for 5min and blocked with 0.4% BSA/PBS for 1h. After rinsing with TBS-T, primary antibodies were bound to the proteins for 1 h and rinsed again with TBS-T; anti-vimentin antibody (1:2000 dilution, V6630, Sigma-aldrich), anti-actin antibody (1:10000 dilution, MAB1501R, Millipore) and anti-GAPDH antibody (1:15000 dilution, 016-25523, Wako) were used. The protein bands were visualized by secondary antibody of anti mouse IgG labeled with HRP (1:1000 dilution, HAF007, R&D) for 5 min and image was obtained by Typhoon (Molecular dynamics). Densitogram was performed with use of image analysis soft ware Image J.

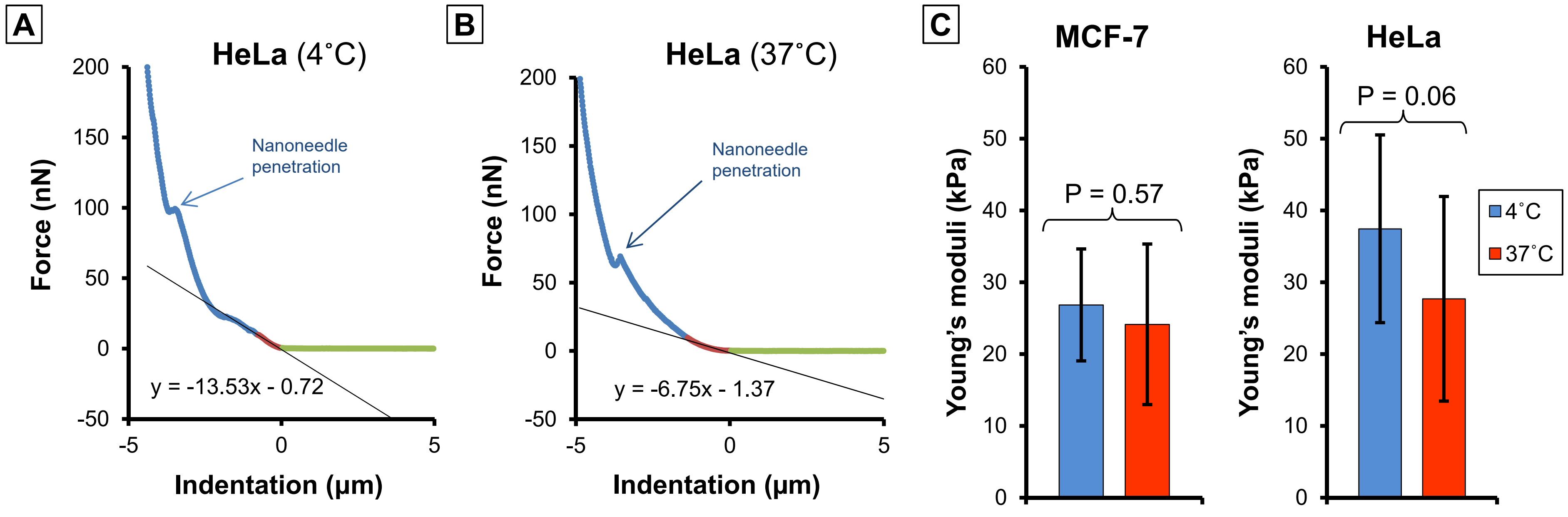

Figure S4. Stiffness of cells measured during nanoneedle insertion at different temperatures. (A) Examples of force-indentation curve during nanoneedle insertion process into HeLa cells at 4˚C and (B) 37˚C. (C) Young's moduli of initial deformation, which is assumed to reflect the stiffness of the plasma membrane, were calculated from fitting to the Hertz model in a defined regime (plotted in red); the regime starts from a point where the slope becomes larger than 0.05 nN/μm and end at force of 10 nN. From fitting to the equation of $F = 2aE/((1-v^2))\ I$, Young's moduli of each cell type at 4˚C or 37˚C were calculated; *F*, *I*, E, *v*, *a* stand for force (nN) applied to cantilever, indentation (μm), Young's modulus (kPa), Poisson's ratio that is assumed to be 0.5, and tip radius of the cantilever (0.1 μm) respectively. Secondary deformation with steeper slope (blue plots) can be attributed to the stiffer structure of the cytoskeletal network, which mainly consists of fibrous actin networks.
